# Risk indicators for oral ulcers among people living with HIV during the first wave of the pandemic: a cross sectional study

**DOI:** 10.1186/s12903-023-03330-2

**Published:** 2023-08-27

**Authors:** Morenike Oluwatoyin Folayan, Roberto Ariel Abeldaño Zuñiga, Jorma I. Virtanen, Nourhan M. Aly, Oliver C Ezechi, Joanne Lusher, Maha El Tantawi, Annie L Nguyen

**Affiliations:** 1 MEHEWE Study Group, Obafemi Awolowo University, Ile-Ife, Nigeria; 2 Department of Child Dental Health, Obafemi Awolowo University, Ile-Ife, Nigeria; 3 Nigeria Institute of Medical Research, Yaba, Lagos, Nigeria; 4 Postgraduate Department, University of Sierra Sur., Oaxaca, Mexico; 5 Faculty of Medicine, University of Bergen, Bergen, Norway; 6 Department of Pediatric Dentistry and Dental Public Health, Faculty of Dentistry, Alexandria University, Alexandria, Egypt; 7 Provost’s Group, Regent’s University London, London, UK; 8 Department of Family Medicine, Keck School of Medicine, University of Southern California, Los Angeles, CA USA

**Keywords:** Viral load, Adherence to antiretroviral therapy, SARS-CoV-2 infection, Co-morbidities, People living with HIV, HIV, COVID-19, Oral ulcers

## Abstract

**Background:**

Little is currently known about HIV-related parameters that may increase the risk for oral ulcers during the COVID-19 pandemic. This study aimed to overcome this gap in research by assessing the associations between HIV viral load, antiretroviral adherence profile, co-morbidity status, SARS-CoV-2 infection and oral ulcers among people living with HIV (PLHIV).

**Methods:**

This was a secondary analysis of data generated from 21,206 to 18 years and above, recruited from 152 countries through an online survey between July and December 2020. Data were extracted for 874 people who reported living with HIV. The dependent variable was reporting having oral ulcer. The independent variables were the viral load, adherence to antiretroviral treatment and a history of SARS-CoV-2 infection. The confounding variables were age at last birthday and sex at birth. A multivariable logistic regression analysis was conducted to determine the associations between the dependent and independent variables after adjusting for the confounding variables.

**Results:**

Of the 874 participants, 99 (11.3%) reported having oral ulcers during the first wave of the COVID-19 pandemic. The odds of PLHIV having oral ulcers during the first wave of the COVID-19 pandemic was significantly higher for people who did not know their viral load than those who had undetectable viral load (AOR: 2.036; 95% CI: 1.204–3.443; p = 0.008); and people who did not adhere to the use of antiretroviral treatment than those who adhered (AOR: 4.113; 95% CI: 2.567–6.589; p < 0.001). Also, PLHIV who had SARS-CoV-2 infection had significantly higher odds of having oral ulcers than those who did not have the infection (AOR: 14.556; 95% CI: 4.500-47.078; p < 0.001). PLHIV who had co-morbidities had non-significantly higher odds of having oral ulcers than those without co-morbidities (AOR: 1.170; 95% CI: 0.656–2.085; p = 0.595).

**Conclusion:**

Oral ulcers may be an indicator of poor adherence to antiretroviral therapy and unsuppressed viral load among PLHIV. It may also be an indicator of SARS-CoV-2 infection and a signal to take prompt and critical care of affected individuals because of the risk for severe COVID-19 for these individuals.

**Supplementary Information:**

The online version contains supplementary material available at 10.1186/s12903-023-03330-2.

## Introduction

Oral ulcers are a pathognomonic feature of COVID-19 [1]. The causative pathway between COVID-19 infection and oral ulcers are both direct and indirect. Directly, it is suggested that severe acute respiratory syndrome coronavirus 2 (SARS-CoV-2) affects the angiotensin-converting enzyme 2 (ACE2), its main host cell receptor, expressed on the epithelial cells of the tongue and of the salivary glands thereby causing oral ulcerations and superficial necrosis [2]. Indirectly, oral ulcers may result from pandemic-related stress. Stress increases the level of salivary cortisol or reactive oxygen species [3], increases the quantity and activity of leukocytes [4] and increases the risk for oral tissue biting or chewing to relieve stress [5]. Emotional distress also increases the risk for oral ulcers [6].

The risk for people living with HIV (PLHIV) to COVID-19 induced pathologies is of major concern. This is because PLHIV, especially those with advanced HIV and those who are not on antiretroviral treatment, have higher rates of co-morbidities and behaviours that are associated with severe COVID-19. These include higher rates of inhalational drug use [7], greater tobacco use [8], and hazardous alcohol use [9, 10] which are factors that increase the risk of SARS-CoV-2 infection [11]. PLHIV also have high rates of cancer [12], cardiovascular disease [13], pulmonary disease [14, 15], obesity, diabetes [16] which are all associated with higher risk for severe COVID-19. Also, PLHIV may have low CD4 + T-cell count, and unsuppressed viral load with higher risk for severe COVID-19 [17]. They have higher frequency of autoimmunity and immune dysregulation [16] as well more medical comorbidities that may increase their risk for oral ulcers [18–23].

Oral ulcers may be an early risk indicator for COVID-19 infection in PLHIV. They appear between 0 and 10 days after the start of COVID-19 symptoms with the most common sites being the tongue [2]. Oral ulcers appear as aphthous-like ulcers in young patients with mild cases of COVID-19 and resembling herpes simplex virus-1 necrotic ulcers in the more severe and immunosuppressed older individuals [24]. There are currently no studies identifying key features of HIV infection that may increase the risk of oral ulcers among PLHIV during the COVID-19 pandemic. PLHIV may be at high risk for COVID-19 pandemic induced-stress oral ulcers due to the elevated C-reactive protein, ferritin, and interleukin-6 [25] that are also associated with oral ulcers [26–28]. There is however, little known about HIV-related parameters that may increase the risk of oral ulcers during the pandemic. This study attempted to address this gap by aiming to determine the associations between HIV viral load, antiretroviral adherence profile, co-morbidity status, SARS-CoV-2 infection and oral ulcers among PLHIV.

## Methods

Ethical approval for the study was obtained from the Human Research Ethics Committee at the Institute of Public Health of the Obafemi Awolowo University Ile-Ife, Nigeria (HREC No: IPHOAU/12/1557). Study participants provided consent before participating in the online survey.

### Study design and study participants

This was a secondary analysis of data generated from 21,206 participants recruited from 152 countries in a cross-sectional study through an online survey between July and December 2020. The details of the parent study, including participants’ recruitment process [29, 30] and the tool used to collect the data [31] had been previously published. The overall Content Validity Index of the questionnaire was 0.83 [31].

Respondents had to be 18 years and above, understand the survey language (survey was conducted in English, French, Arabic, Portuguese and Spanish) and could access the survey using an electronic device and an internet connection. There were no exclusion criteria. The study population were respondents who reported living with HIV by checking a box to indicate that they had HIV on a checklist of 27 medical ailments [32].

### Sample size

The data of 874 participants who indicated they were living with HIV were extracted for analysis. The sample was considered statistically adequate as there was a minimum of 10 participants with complete responses for each of the independent variables in the study. This enabled us to perform regression analysis with a minimum probability level of 0.05 [33].

### Study participants recruitment

This was a non-probability sample of respondents recruited using an online survey tool (Survey Monkey®). Initial participants reached by 45 members of the MEHEWE Study Group (www.mehewe.org) were asked to share the survey link with their contacts around the world. The link to the survey questionnaire was posted on social media groups (Facebook, Twitter, and Instagram), network email lists, and WhatsApp groups. Respondents were encouraged to share the link further with their networks. Details on study participants’ recruitment process had been published [29, 30].

### Study procedures

The survey was preceded by a brief introduction explaining the purpose of the study, assuring participants of their voluntary participation and confidentiality of their data. Before proceeding, participants were required to check a box that indicated consent. Indicating consent was required to take the survey and get access to the study questionnaire. The questionnaire took, on average, 11 min to complete. Multiple best-practice procedures were performed to increase the quality of data collected by the survey. Each participant could only complete a single questionnaire through IP address restrictions. They could edit their answers freely until they chose to submit. Full details of the methodology can be found elsewhere [29, 31].

### Study variables

The dependent variable was reported history of oral ulcer during the pandemic. Participants were asked if they had had oral ulcers during the lockdown with the option responses of ‘yes’ or ‘no’. The method of enquiry about oral ulcers had been reported in prior studies [6, 31].

The independent variables were viral load, adherence to antiretroviral treatment, and a history of SARS-CoV-2 infection. Participants selected response to questions about viral load - What is your HIV viral load (undetectable, detectable and do not know); adherence - Some people find that they sometimes forget to take their medications to manage their HIV. Did you miss any doses of your HIV medications during COVID-19 (Yes, No); and SARS-CoV-2 infection- whether they had tested positive for COVID-19 (Yes, No).

Also, participants living with co-morbidities were identified. All participants who indicated they had any of the 23 listed health conditions presented on a checklist in addition to other health conditions not listed were identified as living with co-morbidities. These included medical conditions which put individuals at higher risk for severe COVID-19 disease (respiratory conditions, diabetes, cancer, heart condition) and those that might put people at moderate risk of COVID-19 disease (respiratory problems, hypertension, depression). The list of health conditions was adopted from Marg et al. [34].

The confounding variables were age at last birthday and sex at birth. Age is associated with viral load suppression and adherence to antiretroviral treatment [35], risk for SARS-CoV-2 infection [36] and risk of oral ulcers [37]. Similarly, sex is associated with viral load [38], adherence to antiretroviral treatment [39], risk for SARS-CoV-2 infection [40] and risk of oral ulcers [41].

### Data analyses

A complete case approach was used, such that observations with missing data were removed from analysis [42]. A multivariable logistic regression analysis was conducted to determine the associations between the dependent and independent variables after adjusting for the confounding variables. Adjusted odds ratios (AOR) and 95% confidence intervals (CI) were calculated. Statistical analysis was conducted using SPSS version 22 and the significance was set at 5%.

## Results

Table 1 shows that of the 874 participants, 99 (11.3%) reported having oral ulcers during the COVID-19 pandemic. Of the 188 (21.5%) participants with detectable viral load, 22 (11.7%) had oral ulcers. Of the 202 (23.1%) participants who had poor adherence to ARV during the pandemic, 54 (26.7%) had oral ulcers. Of the 197 (22.5%) living with co-morbidities, 28 (14.2%) had oral ulcers. Also, of the 18 (2.1) participants who had SARS-CoV-2 infection, 12 (66.7%) had oral ulcers.

The odds of PLHIV having oral ulcers during the COVID-19 pandemic was significantly higher for people who did not know their viral load than those who had undetectable viral load (AOR: 2.036; 95% CI: 1.204–3.443; p = 0.008); and people who did not adhere to the use of antiretroviral than those who adhered (AOR: 4.113; 95% CI: 2.567–6.589; p < 0.001). Also, PLHIV who had SARS-CoV-2 infection had significantly higher odds of having oral ulcers than those who did not have the infection (AOR: 14.556; 95% CI: 4.500-47.078; p < 0.001). The presence of co-morbidities had a non-significant association with having oral ulcers (AOR: 1.170; 95% CI: 0.656–2.085; p = 0.595).

**Table 1:** Multivariable analysis for factors associated with oral ulcers in people living with HIV during the first wave of the pandemic (N = 874)

Variables	Total N = 874 n (%)	Oral ulcers		COR; 95% CI; p value	AOR; 95% CI; p value
		Yes N = 99 (11.3%) n (%)	No N = 775 (88.7%) n (%)		
**Viral load**					
Detectable	188 (21.5)	22 (11.7)	166 (88.3)	1.529; 0.876–2.670; p = 0.135	0.770; 0.396–1.498; p = 0.441
Undetectable	464 (53.1)	37 (8.0)	427 (92.0)		1.000
I don’t know	222 (25.4)	40 (18.0)	182 (82.0)	2.536; 1.570–4.097; p < 0.001	2.036; 1.204–3.443; p = 0.008
**Adherence to ARV**					
Yes	672 (76.9)	45 (6.7)	627 (93.3)		1.000
No	202 (23.1)	54 (26.7)	148 (73.3)	3.992; 2.777–5.739; p < 0.001	4.113; 2.567–6.589; p < 0.001
**Living with HIV and comorbidity**					
Comorbidity	197 (22.5)	28 (14.2)	169 (85.8)	1.414; 0.884–2.261; p = 0.160	1.170; 0.656–2.085; p = 0.595
No comorbidities	677 (77.5)	71 (10.5)	606 (89.5)		1.000
**SARS-CoV-2 infection**					
Yes	18 (2.1)	12 (66.7)	6 (33.3)	17.678; 6.473–48.283; p < 0.001	14.556; 4.500-47.078; p < 0.001
No	856 (97.9)	87 (10.2)	769 (89.8)		1.000
**Mean Age (SD)**	39.2 (10.9)	34.6 (9.3)	39.8 (11.0)	0.954; 0.934–0.974; p = 0.001	0.964; 0.942–0.986; p = 0.001
**Sex at birth**					
Female	492 (56.3)	39 (7.9)	453 (92.1)	0.505; 0.345–0.738; p < 0.001	0.459; 0.286–0.737; p = 0.001
Male	382 (43.7)	60 (15.7)	322 (84.3)		1.000

COR: crude odds ratios; AOR: adjusted odds ratios, CI: confidence interval

## Discussion

The study findings indicate that PLHIV who do not know their viral load, do not adhere to the use of antiretrovirals and who had SARS-CoV-2 infection may have significantly higher likelihood of having oral ulcers during the pandemic. Having a co-morbidity did not seem to be associated with oral ulcers in PLHIV during the pandemic. Though oral ulcers are a common feature of COVID-19, we provide evidence to suggest that there may be differential risk for oral ulcers among PLHIV during the COVID-19 pandemic.

One of the strengths of the study is the population level analysis of the risk indicators of oral ulcers. There are, however, limitations associated with the study. First, this is a cross sectional study and so causal inferences cannot be made. Second, the convenience sampling and use of an online survey, with the unintended exclusion of persons without smartphones and people whose languages were not covered by the study instrument, limit the generalisability of findings. Third, the self-reported parameters are susceptible to recall and social desirability bias [43, 44], moreso the reporting of viral suppression [45], and less so the reporting of adherence [46]. Despite these limitations, the study generated important findings that may need to be explored further.

First, having an unknown viral load is suggestive of poor monitoring of one’s viral load. Reasons why an individual may not know their viral load range from poor access to viral load testing to lack of appropriate action on viral load results especially in low-resource settings [47]. Oral lesions are associated with high viral load among PLHIV [48]. Oral ulcers in PLHIV may therefore be an indicator of a deterioration of the health of the patient [49]. The finding of an association between oral ulcers and poor adherence to the use of antiretroviral therapy may also be a pointer to this. In effect, oral ulcers may indicate decreased cluster-differentiated (CD4+) T cell count and increased viral load and thus, may serve as a pointer to the diagnosis, progression, and prognosis of HIV infection [50].

Second, though very few PLHIV had SARS-CoV-2 infection, the odds of reporting oral ulcers by PLHIV who had SARS-CoV-2 infection was 14 times higher than those who did not report SARS-CoV-2 infection. Prior studies highlighted that PLHIV do not have higher risk of SARS-CoV-2 infection, but once exposed, have higher risk of severe COVID-19 outcomes [50]. The risk of severe outcomes in PLHIV who have SARS-CoV-2 infection is higher in persons with poor adherence to antiretroviral therapy, detectable viral load and with co-morbidities [51].

However, little is currently little understood about the pathophysiology of oral ulcers associated with COVID-19. Oral ulcers may result from the opportunistic infections due to prolonged use of steroids to treat SARS-CoV-2 infection. It also may result from immunosuppression and poor oral hygiene or due to direct infection of the oral tissues [52]. Studies on the pathophysiology of oral ulcers associated with COVID-19 will need to include exploration on the effects of the interactions with HIV as our study suggests that viral loads may interact with SARS-CoV-2 to produce an elevated response in the form of oral ulcers.

In conclusion, although the presence of oral ulcers are a pathognomonic feature of COVID-19, oral ulcer in PLHIV during the COVID-19 pandemic may be an indicator of poor adherence to antiretroviral therapy and unsuppressed viral load as suggested by individuals reporting they do not know their viral load status. It may also be an indicator of SARS-CoV-2 infection and a signal to take prompt and critical care of affected individuals because of the risk of severe COVID-19 for these individuals. Clearly there is scope for more work to be done to further understand these associations.

### Electronic supplementary material

Below is the link to the electronic supplementary material.

Supplementary Material 1

## Data Availability

The datasets used and/or analysed during the current study are available from the corresponding author upon reasonable request.

## Abbreviations

AOR: Adjusted Odds Ratio
CI: Confidence Interval
COVID-19: Coronavirus infectious disease 2019
HIV: Human Immunodeficiency Virus
